# The role of sleepiness on arterial stiffness improvement after CPAP therapy in males with obstructive sleep apnea: a prospective cohort study

**DOI:** 10.1186/s12890-017-0518-z

**Published:** 2017-12-08

**Authors:** Maria Alexandra Mineiro, Pedro Marques da Silva, Marta Alves, Ana Luísa Papoila, Maria João Marques Gomes, João Cardoso

**Affiliations:** 1 Pulmonology Department, Centro Hospitalar de Lisboa Central, Lisbon, Portugal; 2 Arterial Investigation Unit, Internal Medicine Department IV, Centro Hospitalar de Lisboa Central, Lisbon, Portugal; 3 Epidemiology and Statistics Unit, Research Centre, Centro Hospitalar de Lisboa Central, Lisbon, Portugal; 4 NOVA Medical School / Faculdade de Ciências Médicas, Lisbon, Portugal

**Keywords:** Obstructive sleep apnea, Daytime sleepiness, Arterial stiffness, Carotid-femoral pulse wave velocity

## Abstract

**Background:**

Obstructive sleep apnea (OSA) is associated with increased cardiovascular risk. This study aim to assess differences in changes in arterial stiffness of two groups of patients, defined as having daytime sleepiness or not, after continuous positive airway pressure (CPAP) treatment.

**Methods:**

A selected cohort of consecutive male patients, under 65 years old, with moderate to severe OSA and without great number of comorbidities was studied. The diagnosis was confirmed by home respiratory poligraphy. Sleepiness was considered with an Epworth Sleepiness Scale (ESS) > 10.

An ambulatory blood pressure (BP) monitoring and carotid-femoral pulse wave velocity (cf-PWV) measurements were performed, before and after four months under CPAP. Compliant patients, sleepy and non-sleepy, were compared using linear mixed effects regression models. A further stratified analysis was performed with non-sleepy patients.

**Results:**

Thirty-four patients were recruited, with mean age 55.2 (7.9) years, 38.2% were sleepy, 79.4% with hypertension, 61.8% with metabolic syndrome and 82.4% with dyslipidaemia.

In univariable analysis, cf-PWV was strongly related to systolic BP parameters and age, but also to antihypertensive drugs (*p* = 0.030), metabolic syndrome (*p* = 0.025) and daytime sleepiness (*p* = 0.004). Sleepy patients had a more severe OSA, with AHI 44.8 (19.0) vs 29.7 (15.7) events/h (*p* = 0.018), but sleep study parameters were not associated with cf-PWV values. On multivariable regression, a significant interaction between time (CPAP) and sleepiness (*p* = 0.033) was found.

There was a weak evidence of a cf-PWV reduction after CPAP treatment (*p* = 0.086), but the effects of treatment differed significantly between groups, with no changes in non-sleepy patients, while in sleepy patients a significant decrease was observed (*p* = 0.012).

Evaluating non-sleepy patients group under CPAP therapy, results showed that both higher pulse pressure (*p* = 0.001) and lower LDL-cholesterol levels (*p* = 0.015) at baseline were associated to higher cf-PWV changes.

**Conclusions:**

Patients with daytime sleepiness had a more severe OSA and presented a greater arterial stiffness improvement after CPAP therapy, independently from age and BP. Besides sleepiness, cf-PWV reduction after CPAP therapy was mainly associated to CV risk factors, and less to sleep study parameters.

**Trial registration:**

Clinicaltrials.gov NCT02273089 23.10.2014 retrospectively registered.

**Electronic supplementary material:**

The online version of this article (10.1186/s12890-017-0518-z) contains supplementary material, which is available to authorized users.

## Background

Obstructive sleep apnea (OSA) is a disorder characterized by repeated episodes of upper airway collapse during sleep [1]. OSA is independently associated with several cardiovascular (CV) diseases including hypertension, myocardial infarction, and stroke [2]. The effects of OSA during sleep, and especially intermittent hypoxia, may have a predominant role in the vascular consequences of OSA, in association with other mechanisms like oxidative stress, systemic inflammation, impaired glucose and lipid metabolism, endothelial dysfunction and sympathetic activation [3]. The early detection of individuals at higher risk for CV disease and their treatment is a priority [4].

The condition of the arterial vessels can be studied by pulse wave velocity (PWV), that can be used as an index of arterial distensibility. Arterial PWV describes how fast a blood pressure (BP) pulse travels from one point to another in an artery. Arterial stiffness is now recognized as a major driver of CV disease, and it depends mainly on age and BP. PWV is a well-established procedure for measuring the arterial stiffness, using arterial tonometry, at the carotid and femoral artery sites: carotid-femoral pulse wave velocity (cf-PWV), the gold standard index. It is a noninvasive biomarker for primary and secondary CV disease prevention, and can be used as an intermediate endpoint for CV events [5].

OSA is associated with increased arterial stiffness, with an improvement after continuous positive airway treatment (CPAP) [6, 7].

However, most of OSA patients remain undiagnosed. Daytime sleepiness, a symptom of OSA easily identifiable, was reported in about 20% of all adults with an Apnea-Hypopnea Index (AHI) ≥ 5/h, in population based studies [8, 9]. Daytime sleepiness does not correlate well with OSA severity parameters and, in its absence, the indication for treatment of OSA is still debatable.

Although non-sleepy patients have an augmented arterial stiffness when compared to non-OSA controls [10], the very few studies performed did not find a significant improvement after CPAP. Studies that assessed CV risk did not find any benefit either [11].

The aim of this study is to verify if there are differences in the progression of arterial stiffness in a cohort of patients with OSA, classified as sleepy and non-sleepy, undergoing treatment with CPAP for four months.

## Methods

### Study design and patient recruitment

This is a prospective cohort study conducted between October 2012 to November 2015. Consecutive patients referred for snoring or OSA-related symptoms to the Sleep Outpatient Clinic of a Pneumology Department were recruited if they were male, younger than 65 years old, and with moderate to severe OSA (AHI > 15/h) [1].

The exclusion criteria included other sleep disorders (clinically identified); alcohol abuse; known CV disease beyond hypertension; and other chronic diseases.

Patients with high levels of fasting glucose performed an additional oral glucose tolerance test, and excluded if they were diabetic.

Patients were evaluated monthly for assessment of effectiveness and compliance to therapy, presence of nasal complaints, and changes on the equipment. They were excluded from the study if they were not compliant to CPAP (using at least mean of 4 h/night) [12], if significant weight loss (> 5%) occurred, or if there was introduction of new drugs or emergence of new comorbidities.

A laboratorial evaluation, ambulatory blood pressure monitoring (ABPM) and cf-PWV measurements were made before and after 4 months of the beginning of CPAP therapy.

ABPM was performed with Ambulatory Blood Pressure - SpaceLabs model 90207®. Patients were considered hypertensive according ABPM values (current guidelines) or when they were under antihypertensive medication.

At baseline consultation, the Epworth Sleepiness Scale (ESS) was applied and patients were classified as sleepy (ESS > 10) or non- sleepy (ESS ≤ 10) [13]. The comorbidities and drug treatments were registered.

A detailed operational definition and description of the primary and secondary outcomes assessment can be found in our previous paper [14]. For ethical reasons, each patient will act as its own control. The study was approved by the Ethics Committees of our hospital center (reference number 84/2012) and NOVA Medical School/ Faculdade de Ciências Médicas da Universidade Nova de Lisboa (number 36/2014/CEFCM). Written informed consent was obtained from all patients.

### Sleep study and CPAP titration

The diagnosis of OSA was confirmed by home respiratory poligraphy (Embletta® system, Broomfield, USA), which includes continuous recording from nasal cannulae (pressure and flow), thoracic-abdominal motion, pulse oximetry, electrocardiogram and body position sensor. Respiratory events were classified using standard criteria [15].

All patients performed CPAP titration using an automatic auto-CPAP device (ResMed S9 AutoSet, California, USA) for 3 nights, and subsequent change to CPAP [16]. After downloading the data from CPAP’s card, the definite value of CPAP was the amount of pressure that eliminates events in 95% of sleep time.

### Carotid-femoral pulse wave velocity (cf-PWV) study

Assessment of cf-PWV was made using a non-invasive automatic device (Complior®, Colson, Paris). Measurements were performed in the morning, without prior consumption of tea or coffee. The assessment was performed in the supine position, and the values ​​took into account the concomitant arterial BP [5].

CF-PWV was measured using the «foot-to-foot» velocity method from the pressure waveforms, obtained using surface tonometry probes at the right common carotid artery and the right femoral artery. The time delay (Dt, or transit time) is measured between the «foot» of these two waveforms. The distance D covered by the waves is incorporated to the skin distance the two tonometry probes, i.e. the common carotid artery and the common femoral artery. PWV is estimated as PWV = D/Dt (m/s) [5].

### Statistical analysis

Patients’ characteristics were described using the mean and standard deviation (SD) for continuous variables, and frequencies (percentages) for categorical variables. To compare characteristics of patients before and after CPAP therapy, Wilcoxon signed rank test and McNemar test were used. In this analysis, demographic variables, sleep study parameters, blood tests and vascular parameters were used. Additionally, in order to investigate associations between these variables and cf-PWV, linear mixed effects regression models were applied. All the variables that attained a *p*-value ≤0.25 were considered for the multivariable analysis. As in this analysis an interaction was identified between ESS group and time (defined by the two assessment instants: before and after CPAP), a stratified analysis by ESS group was also performed in order to investigate potential associations between baseline variables and changes in cf-PWV (∆cf-PWV = cf-PWV before CPAP- cf-PWV after CPAP). In this stratified analysis, linear regression models were only used in the non-sleepy group, attending to the small sample size of sleepy patients’ group.

The level of significance α = 0.05 was considered. All data were analyzed using the Statistical Package for the Social Sciences for Windows 21.0 (IBM Corp. Released 2012. IBM SPSS Statistics for Windows. Armonk, NY: IBM Corp.) and Stata (StataCorp. 2013. Stata Statistical Software: Release 13. College Station, TX: StataCorp LP.).

## Results

Forty-two patients with moderate to severe OSA were recruited for this study.

Three patients refused the treatment and five patients dropped out later due to poor compliance to CPAP treatment.

The final cohort included 34 patients, 13 (38.2%) patients with daytime sleepiness, 19 (55.9%) patients with moderate OSA and 15 with severe OSA. Clinical characteristics of patients and home respiratory poligraphy data are shown in Table 1.

**Table 1:** Demographic and clinical characteristics of patients before and after CPAP therapy (*n* = 34)

Variables	Before CPAP	After CPAP	*p*-value
Age	55.2 (7.9)	–	–
BMI	31.2 (4.1)	31.3 (4.0)	0.901
Smoking	7 (20.6)	–	–
ESS >10	8.4 (4.3)	4.8 (2.7)	<0.001^*^
AHI (events/h)	35.2 (18.8)	2.6 (2.3)	<0.001
ODI (events/h)	25.5 (20.0)	–	–
Mean SaO_2_ (%)	92.1 (2.1)	–	–
Lower SaO_2_ (%)	79.8 (8.5)	–	–
SaO_2_ < 90% (%)	17.7 (18.5)	–	–
Total cholesterol, mg/dl	198.2 (32.9)	187.4 (36.0)	0.021
HDL-cholesterol, mg/dl	44.3 (10.3)	44.5 (11.0)	0.678
LDL-cholesterol, mg/dl	131.0 (29.4)	121.5 (30.8)	0.031
Triglycerides, mg/dl	137.9 (51.0)	126.6 (59.6)	0.124
Glucose, mg/dl	98.7 (12.8)	98.2 (11.7)	0.924
HbA1c, %	5.7 (0.4)	5.8 (0.4)	0.064
24 h SBP (mm Hg)	129.8 (10.5)	128.0 (10.3)	0.287
24 h DBP (mm Hg)	82.4 (7.1)	79.2 (6.4)	0.004
Pulse pressure (mm Hg)	47.4 (7.3)	48.8 (8.1)	0.050
24 h MBP (mm Hg)	97.4 (7.9)	94.9 (7.2)	0.030
Day-time SBP (mm Hg)	133.3 (10.5)	131.2 (10.9)	0.214
Day-time DBP (mm Hg)	85.4 (7.3)	81.6 (6.9)	0.001
Night-time SBP (mm Hg)	117.9 (12.6)	116.4 (9.5)	0.363
Night-time DBP (mm Hg)	72.1 (9.1)	70.1 (5.9)	0.169
Hypertension n (%)	27 (79.4)	25 (73.5)	0.500^*^
Antihypertensive drugs, n (%)	21 (61.8)	–	–
Dyslipidaemia n (%)	28 (82.4)	24 (70.6)	0.125^*^
Lipid lowering drugs n (%)	14 (41.2)	–	–
MetS n (%)	21 (61.8)	20 (58.8)	1.000^*^
Cf-PWV (m/s)	12.3 (2.3)	11.7 (1.8)	0.086

Results are expressed as mean (standard deviation); *p-values obtained by McNemar test; remaining *p*-values were obtained by Wilcoxon rank exact test

*CPAP* continuous positive airway treatment, *BMI* Body mass index, *ESS* Epworth Sleepiness Scale, *AHI* Apnea/Hypopnea Index, *ODI* Oxygen Desaturation Index, *SaO*_*2*_ arterial oxygen saturation, *HbA1c* glycated haemoglobin, *SBP* systolic blood pressure, *DBP* diastolic blood pressure, *MetS* metabolic syndrome, cf*-PWV* carotid-femoral pulse wave velocity

Most patients (27; 79.4%) were hypertensive; 6/27 (22.2%) had no treatment and 21/27 (77.8%) were under antihypertensive medication. In 14/27 (51.9%) patients BP was uncontrolled; 10/21 (47.6%) patients were on monotherapy, 7/21 (33.3%) and 4/21 (19.0%) patients were on two or three antihypertensive drugs, respectively. Dyslipidaemia was highly prevalent, as well as metabolic syndrome (MetS) [17].

### Effects of CPAP

After four months of CPAP, there was a significant decrease of ESS (*p* ≤ 0.001). Regarding AHI, there was also a significant decrease (*p* < 0.001) (Table 1), with a mean compliance to CPAP treatment of 5.7 (1.1) hours, and a mean pressure of CPAP 9.3 (0.2).

There was an improvement in some metabolic parameters, namely total cholesterol (*p* = 0.021) and LDL-cholesterol (*p* = 0.031). After treatment with CPAP there was a non-significant decrease in dyslipidaemia prevalence (*p* = 0.125) (Table 1).

There was also an improvement in BP parameters, with a decrease in 24 h diastolic BP (DBP) (*p* = 0.004), daytime DBP (*p* = 0.001) and 24 h mean BP (MBP) (*p* =  0.030), and with an increase in pulse pressure (*p* = 0.050). Non-dipping prevalence in 24 h BP evaluation decreased from 7 (20.6%) to 5 (14.7%) patients, without significance (*p* = 0.500).

Also, a weak evidence of a decrease of cf-PWV values was found (*p* = 0.086) (Table 1). Although without statistical significance, the prevalence of normal cf-PWV (<10 m/s) increased from 5/34 (14.7%) to 9/34 (26.5%) patients (*p* = 0.289).

### Parameters related to cf-PWV

In univariable analysis there was an association between age, ESS, 24 h SBP, daytime SBP, night-time SBP, pulse pressure, the use of antihypertensive drugs and the presence of MetS with higher values of cf-PWV (Table 2).

**Table 2:** Univariable regression analysis for cf-PWV

Variables	Coefficient estimate (95% CI)	*p*-value
Time	−0.52 (−1.06; 0.02)	0.057
Age	0.11 (0.04; 0.02)	0.002
BMI	0.01 (−0.14; 0.17)	0.899
Smoking	−0.47 (−2.05; 1.11)	0.561
ESS	0.13 (0.04; 0.23)	0.004
ESS >10	−0.15 (−1.47; 1.17)	0.822
AHI (events/h)	0.01 (−0.03; 0.04)	0.725
ODI (events/h)	0.01 (−0.03; 0.04)	0.705
Mean SaO_2_ (%)	−0.06 (−0.37; 0.26)	0.730
Lower SaO_2_ (%)	0.04 (−0.04; 0.11)	0.366
SaO_2_ < 90% (%)	−0.002 (−0.04; 0.03)	0.917
CPAP compliance	−0.09 (−0.67; 0.48)	0.752
Total cholesterol, mg/dl	−0.002 (−0.02; 0.02)	0.794
HDL-cholesterol, mg/dl	−0.05 (−0.10; 0.01)	0.127
LDL-cholesterol, mg/dl	−0.01 (−0.03; 0.01)	0.554
Triglycerides, mg/dl	0.01 (−0.00; 0.02)	0.168
Glucose, mg/dl	0.02 (−0.01; 0.05)	0.229
HbA1c, %	0.41 (−0.076; 1.59)	0.490
24 h SBP (mm Hg)	0.09 (0.04; 0.14)	<0.001
24 h DBP (mm Hg)	0.05 (−0.01; 0.12)	0.109
Pulse pressure (mm Hg)	0.15 (0.08; 0.21)	<0.001
24 h MBP (mm Hg)	0.04 (−0.02; 0.10)	0.224
Day-time SBP (mm Hg)	0.09 (0.04; 0.13)	<0.001
Day-time DBP (mm Hg)	0.04 (0.02; 0.11)	0.167
Night-time SBP (mm Hg)	0.07 (0.03; 0.11)	0.002
Night-time DBP (mm Hg)	0.05 (−0.01; 0.11)	0.109
Hypertension	1.29 (−0.24; 2.81)	0.099
Antihypertensive drugs	1.37 (0.13; 2.61)	0.030
Dyslipidaemia	0.49 (−1.19; 2.17)	0.569
Lipid lowering drugs	0.22 (−1.09; 1.52)	0.745
MetS	1.44 (0.18; 2.70)	0.025

*CPAP* continuous positive airway treatment, *BMI* Body mass index, *ESS* Epworth Sleepiness Scale, *AHI* Apnea/Hypopnea Index, *ODI* Oxygen Desaturation Index, *SaO*_*2*_ arterial oxygen saturation, *HbA1c* glycated haemoglobin, *SBP* systolic blood pressure, *DBP* diastolic blood pressure, *MetS* metabolic syndrome, cf*-PWV* carotid-femoral pulse wave velocity, p-values were obtained by linear mixed effects regression models

Relating to the group of patients with MetS and comparing to the patients without MetS, there was a higher prevalence of hypertension (*p* = 0.004) and antihipertensive drugs (*p* < 0.001). Triglycerides (*p* = 0.002) and glucose (*p* = 0.003) levels were higher, but presented lower HDL-cholesterol levels (*p* = 0.004) and lower mean SaO_2_ (*p* = 0.002).

Moreover, levels of cf-PWV were higher than in patients without MetS, both at baseline (*p* = 0.04) and follow up (*p* = 0.04).

Results of the multivariable regression model showed an association between ESS group and cf-PWV after adjusting by age, pulse pressure and time.

**Fig. 1:**
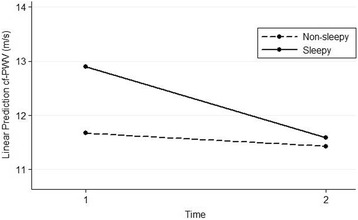
Interaction between ESS group and time regarding cf-PWV measurements. Time: 1-before CPAP; 2- after CPAP. Sleepy group: ESS > 10; non-sleepy group: ESS ≤ 10. ESS- Epworth Sleepiness Scale

The final multivariable linear mixed regression model (Table 3), after taking into account the coefficient estimate of the interaction term, revealed a different effect of CPAP in the two groups of patients, with a more significant reduction from baseline in cf-PWV in sleepy (1.31 m/s) than in non-sleepy (0.24 m/s) patients. Regarding ESS group, still considering the coefficient estimate of the interaction term, a higher cf-PWV difference between the two groups exists at baseline (1.23 m/s) when compared to four months later values (0.15 m/s) (Fig. 1).

**Table 3:** Multivariable regression analysis for cf-PWV

Variables	Coefficient estimate (95%CI)	*p*-value
Age	0.07 (0.01; 0.13)	0.015
Time	−0.24 (−0.89; 0.41)	0.470
Pulse pressure (mm Hg)	0.16 (0.10; 0.21)	<0.001
ESS >10	1.23 (0.18; 2.27)	0.022
Time×ESS*	−1.07 (−2.05; −0.09)	0.033

*ESS* Epworth Sleepiness Scale; *interaction term of ESS group and time (defined by the two assessment instants: before and after CPAP); *p*-values were obtained by linear mixed effects regression models

Moreover, for each increase of 1 year of age there was a cf-PWV mean increase of 0.07 m/s, and for each mm Hg increase in pulse pressure there was a cf-PWV mean increase of 0.16 m/s.

### Stratified analysis by sleepy and non-sleepy patients

At baseline, sleepy patients had higher AHI than their counterparts, with 44.8 (19) events/h vs 29.7 (15.7) events/h (*p* = 0.018). No differences were found in age, BMI, or metabolic parameters between these two groups. There was also no difference in the prevalence rates of BP, antihipertensives or lipid lowering drugs (Table 4).

**Table 4:** Demographic and clinical characteristics of patients before and after CPAP therapy by ESS group

	Sleepy (*n* = 13)		Non-sleepy (*n* = 21)	
Variables	Before CPAP	After CPAP	Before CPAP	After CPAP
Age	55.08 (6.2)	–	55.19 (9.0)	–
BMI	31.5 (3.6)	31.1 (3.4)	31.1 (4.4)	31.3 (4.5)
Smoking, n (%)	2 (15.3%)	–	5 (23.8%)	–
ESS^#^	13 (1.8)^*^	5.9 (3.3)^*^	5.5 (2.4)^*^	4.1 (2.0)^*^
AHI (events/h)^#^	44.8 (19.0)^*^	4.0 (3.0)^*^	29.7 (15.7)^*^	1.9 (1,2)^*^
ODI (events/h)^#^	35.8 (21.4)	–	19.2 (16.6)	–
Mean SaO_2_ (%)	91.2 (2.4)	–	92.6 (1.7)	–
Lower SaO_2_ (%)^#^	71 (7.6)	–	77.1 (8.3)	–
SaO_2_ < 90% (%)^#^	26.9 (22.8)	–	12.1 (12.7)	–
Total chol, mg/dl	199.4 (34.0)	188.4 (42.5)	200.5 (34.3)	186.5 (30.5)
HDL-cholesterol, mg/dl	44.7 (12.0)	44.8 (12.1)	44.0 (9.1)	44.3 (10,3)
LDL-cholesterol, mg/dl	128.3 (31.6)	122.5 (33.1)	135.4 (28.6)^*^	120.6 (30.0)^*^
Triglycerides, mg/dl	117.7 (37.7)	120.1 (64.4)	154.3 (56.0)	132.1 (56.9)
Glucose, mg/dl	101.4 (11.8)	97.2 (13.9)	96.8 (13.1)	98.9 (10.2)
HbA1c, %	5.7 (0.4)	5.9 (0.4)	5.7 (0.4)	5.8 (0.3)
24 h SBP (mm Hg)	128.8 (9.8)	125.0 (9.2)	130.6 (11.2)	130.3 (10.8)
24 h DBP (mm Hg)	83.7 (7.7)^*^	78.7 (5.9)^*^	81.5 (6.7)	79.6 (6.9)
Pulse pressure (mm Hg)	45.2 (4.6)	46.3 (6.9)	49.1 (8.6)	50.7 (8.7)
24 h MBP (mm Hg)	97.9 (8.5)^*^	92.8 (6.4)^*^	97.1 (7.6)	96.4 (7.5)
Dt SBP (mm Hg)	133.3 (9.4)	127.6 (9.5)	134.8 (11.6)	133.9 (11.3)
Dt DBP (mm Hg)	86.5 (7.8)^*^	80.9 (6.4)^*^	84.5 (7.1)^*^	82.1 (7.4)^*^
Nt SBP (mm Hg)	117.8 (11.9)	116.7 (9.5)	118.0 (13.4)	116.1 (9.8)
Nt DBP (mm Hg)	74.1 (9.2)	71.0 (5.7)	70.7 (9.1)	69.4 (6.2)
Hypertension, n (%)	11 (84.6%)	–	16 (76.2%)	–
Hypertensive drugs, n (%)	8 (61.5%)	–	13 (61.9%)	–
Dyslipidemia, n (%)	11(84.6%)	–	17 (81.0%)	–
Lipid lowering drugs, n (%)	5 (38.5%)	–	9 (42.9%)	–
MetS n (%)	8 (61.5%)	–	14 (66.7%)	–
cf-PWV (m/s)	12.5 (1.9)^*§^	11.3 (1.6)^*^	12.1 (2.6)^£^	12.04 (2.0)

Results are expressed as mean (standard deviation); *ESS* Epworth Sleepiness Scale, *AHI* apnea/hypopnea index, *ODI* oxygen desaturation index, *SaO*_*2*_ arterial oxygen saturation, *SaO*_*2*_ *< 90%* time under 90%, *HDL* high density lipoprotein, *LDL* low density lipoprotein, *SBP* systolic blood pressure, *DBP* diastolic blood pressure, *Dt* day-time, *Nt* night-time, *MetS* metabolic syndrome, cf*-PWV* carotid-femoral pulse wave velocity; ^#^ *p* < 0.05 comparison between sleepy and non-sleepy groups, at baseline (Mann-Whitney test); ^*^ *p* < 0.05 comparison between before and after CPAP, in each ESS group (Wilcoxon signed rank test), for cf-PWV (m/s): §*p* = 0.012; £*p* = 0.779, in sleepy and non-sleepy group, respectively

After treatment with CPAP, and regarding cf-PWV, there was a significant decrease in sleepy patients (*p* = 0.012) and a non-significant decrease in non-sleepy patients (*p* = 0.779).

In non-sleepy patients, the change in cf-PWV after four months of CPAP was associated most strongly with pulse pressure (coefficient estimate = 0.08 m/s; 95% CI, 0.01 to 0.15, *p* = 0.034) (Additional file 1).

Also, there was a weak association between cf-PWV change with triglycerides (coefficient estimate = 0.01 m/s; 95% CI, −0.00 to 0.03, *p* = 0.080), and with the presence of lipid lowering drugs (coefficient estimate = 1,23 m/s; 95% CI, −0.09 to 2.56, *p* = 0.066).

To identify the associated variables with cf-PWV changes between baseline and after four months of CPAP therapy, multiple linear regression analysis was performed, revealing that higher pulse pressure was associated with higher cf-PWV changes and higher LDL-cholesterol levels were associated with lower cf-PWV changes (Table 5).

**Table 5:** Results of multivariable model corresponding to cf-PWV changes among 21 patients without daytime sleepiness

Variables	Coefficient estimate (95%CI)	*p*-value
Pulse pressure (mm Hg)	0.13 (0.07; 0.19)	0.001
LDL-cholesterol (mm Hg)	−0.03 (−0.05; −0.01)	0.015

*LDL* low density lipoprotein. Overall model adjusted R^2^ = 0.71

## Discussion

This study is the first to attempt to identify characteristics of sleepy and non-sleepy patients that might justify the different effects of CPAP on arterial stiffness.

Selecting a cohort of male patients with moderate to severe OSA and without a great number of comorbidities, we found a high prevalence of hypertension, dyslipidaemia and MetS.

After four months of CPAP treatment there was an improvement in 24 h DBP, daytime DBP, and levels of total cholesterol. Age and SBP were variables associated to cf-PWV, as expected, but daytime sleepiness was also detected. In multivariable analysis, we found that sleepy and non-sleepy groups behaved differently, under CPAP treatment. In the fitted model, sleepiness was an independent determinant of the longitudinal decrease in cf-PWV (*p* = 0.033 for interaction with time), after adjusting for covariates, meaning time, age and pulse pressure.

Analysing the subgroup of non-sleepy patients, a high pulse pressure and lower levels of LDL cholesterol were predictive of higher cf-PWV changes, in a model where 71% of cf-PWV variability was explained by those independent variables.

The strengths of our study settled, thus, in the use of a standardized marker of vascular disease and stiffness (cf-PWV), and the evaluation of CPAP compliant patients.

There are at least two meta-analysis [6, 7] identifying a decrease in arterial stiffness after CPAP treatment in patients with OSA, and in patients with OSA and hypertension; only four from the selected studies evaluated the effect on cf-PWV.

The effect of CPAP reducing cf-PWV was well described in studies with highly selected samples, including very severe and younger OSA patients [18, 19], or less obese patients [20] than those that we recruited, and this is due to the absence of comorbidities, namely hypertension. Also, in those studies patients were mainly symptomatic and sleepy.

Kohler et al. [21] studied 208 patients less symptomatic, with ESS 8.4 (4), and found that, after six months of CPAP, arterial stiffness (measured by AIx) did not improve, compared to controls. However, patients included in that study had a milder OSA than ours and the median compliance of CPAP was 2.8 h per night. Moreover, in the subgroup with higher compliance there were not significant changes in arterial stiffness, either.

In contrast with these results, in a meta-regression analysis examining the effect of potential modifiers upon the effect of CPAP on arterial stiffness, the sleepiness score did not have significance [6].

We admit that the role of sleepiness as well as the usefulness of cf-PWV measurements may be best defined in this sample under 60 years old and without a great number of comorbidities, yet very prevalent in the clinical set.

Concerning the improvement in lipid profile, our results are in agreement with the meta-analysis results of Nadeem et al. CPAP treatment seems to improve dyslipidaemia, although we did not find an increase in HDL-cholesterol as described [22].

MetS was associated to higher levels of cf-PWV, but it was not included in the multivariable regression model. Patients with MetS were found to have significantly higher baseline and follow up levels of cf-PWV than patients without MetS, besides a more severe OSA defined by lower mean SaO_2_ levels. In fact, MetS is an additive factor to arterial wall damage and only in very selected populations it has been possible to demonstrate a reduction of some of the syndrome components or its prevalence [23].

We found, among the variables selected, that pulse pressure was positively related to cf-PWV values, as well as SBP parameters. In the non-sleepy group analysis pulse pressure was predictive of greater cf-PWV changes, and other BP variables were excluded from the model.

The predominant association of pulse pressure with cf-PWV variations is interesting as it is probably related to early vascular ageing of OSA patients. Pulse pressure has showed the strongest association with aortic cf-PWV over other haemodynamic BP parameters, namely from age 60 years and over, where it begins to increase precipitously. In OSA patients, Sanner et al. also found that a higher pulse pressure before treatment with CPAP was predictive of a beneficial CPAP effect on BP, over other BP parameters [24].

In the sleepy group of patients and after adjusting for age and pulse pressure, there was a reduction of cf-PWV of 1.31 m/s from baseline. Also, and though there was no difference between groups, we found a difference among them of 0.15 m/s, after CPAP therapy.

This is relevant as an increase in aortic PWV by 1 m/s is known to correspond to an age-, sex-, and risk factor-adjusted risk increase of 14%, 15%, and 15% in total CV events, CV mortality, and all-cause mortality, respectively [25].

Patients with daytime sleepiness tend to experience more severe OSA [26], and the severity of OSA could influence the results of CPAP therapy. In this study, the group of sleepy patients has higher AHI, ODI and SaO_2_ < 90% than the non-sleepy patients. However, sleep study parameters were not correlated to cf-PWV changes, neither predictive of a better outcome in non-sleepy group of patients.

The issue of sleepiness as an independent variable was raised in studies on OSA and hypertension [27]. CPAP significantly reduces BP in patients with OSA but with a low effect size [28]. When non-sleepy/less symptomatic patients were evaluated, a DBP reduction of less than 1 mmHg was found in compliant patients and may be significant, but its clinical meaning is debatable [11]. Daytime sleepiness was also identified as independent variable predicting BP changes in patients with OSA under CPAP [29].

In our study, non-sleepy patients had a mean compliance of 5.9 (0.22) hours, and a significant reduction of 2.4 mmHg occurred in daytime DBP.

Regarding the mechanisms involved in the pathophysiology of daytime sleepiness, some studies suggest that it is related to sleep fragmentation [30], while others have found an association with both nocturnal hypoxemia [31] and changes produced by intermittent hypoxemia.

One possible explanation for our findings is based in the knowledge that daytime sleepiness is related to both the systemic inflammation associated with sleep disruption (sleep fragmentation and arousals) and to the severity of nocturnal desaturation, which appears to be the primary proatherogenic feature of OSA [32]. It may thus represent the global effect of OSA severity factors.

### Study limitations

Limitations of this study were the small sample size, though it was not an obstacle for the general aim, and the lack of a control group, which makes this study mainly hypothesis generating. The results cannot be extended to women, the elderly or patients with comorbidities. The use of polysomnography instead of home respiratory poligraphy would allow a more complete evaluation of associations between sleep variables and cf-PWV changes, although it was time consuming and uncomfortable for patients with active working life.

## Conclusions

In a cohort of consecutive middle aged men with moderate to severe OSA, mainly with metabolic syndrome and dyslipidaemia, it was found a significant decrease in cf-PWV, in sleepy but not in non-sleepy patients. In non-sleepy patients, pulse pressure and LDL-cholesterol at baseline were predictive of higher cf-PWV changes. Further studies are needed to confirm the use of cf-PWV as a biomarker of response to CPAP therapy in OSA patients with sleepiness and good compliance to the treatment.

## Additional file

Additional file 1: Table S1. Univariable regression analysis investigating associations between variables and ∆cf-PWV, in non-sleepy patients. (DOCX 13 kb)

## Abbreviations

AHI: Apnea-hypopnea index
BP: blood pressure
Cf-PWV: carotid-femoral pulse wave velocity
CPAP: continuous positive airways pressure
CV: cardiovascular
DBP: diastolic blood pressure
ESS: Epworth Sleepiness Scale
HDL: high-density lipoprotein
LDL: low density lipoprotein
OSA: obstructive sleep apnea
SBP: systolic blood pressure
