# Supplementary material for: The role of sleepiness on arterial stiffness improvement after CPAP therapy in males with obstructive sleep apnea: a prospective cohort study
Source: BMC Pulm Med. 2017 Dec 8;17:182. doi: 10.1186/s12890-017-0518-z (PMC5723049; doi:10.1186/s12890-017-0518-z)
Supplement: Additional file 1: Table S1. — Univariable regression analysis investigating associations between variables and ∆cf-PWV, in non-sleepy patients. (DOCX 13 kb) [file 12890_2017_518_MOESM1_ESM.docx]

| Supplemental Table 1 – Univariable regression analysis investigating associations between variables and ∆cf-PWV, in non-sleepy patients | | |
| --- | --- | --- |
|  | Coefficient estimate (95%CI) | p-value |
| Age | 0.00 (-0.08; 0.08) | 0.999 |
| BMI | -0.04 (-0.21; 0.12) | 0.590 |
| Smoking binary | 0.30 (-1.38; 1.98) | 0.713 |
|  |  |  |
| AHI (events/hour) | -0.01 (-0.05; 0.04) | 0.752 |
| ODI (events/hour) | 0.01 (-0.04; 0.05) | 0.667 |
| Mean SaO2 (%) | -0.14 (-0.56; 0.28) | 0.499 |
| Lower SaO2 (%) | -0.05 (-0.14; 0.03) | 0.206 |
| SaO2<90% (%) | 0.01 (-0.05; 0.07) | 0.744 |
| CPAP pressure | 0.26 (-0.33 0.86) | 0.368 |
|  |  |  |
| Total cholesterol, mg/dl | -0.02 (-0.04; 0.01) | 0.276 |
| HDL-cholesterol, mg/dl | -0.02 (-0.13; 0.08) | 0.632 |
| LDL-cholesterol, mg/dl | -0.02 (-0.05; 0.01) | 0.203 |
| Triglycerides, mg/dl | 0.01 (-0.00; 0.03) | 0.080 |
| Glucose, mg/dl | -0.02 (-0.05; 0.01) | 0.223 |
| HbA1c, % | -0.56 (-2.60; 1.47) | 0.562 |
|  |  |  |
| 24h SBP (mm Hg) | 0.04 (-0.02; 0.10) | 0.177 |
| 24h DBP (mm Hg) | -0.02 (-0.13; 0.09) | 0.761 |
| Pulse pressure (mm Hg) | 0.08 (0.01; 0.15) | 0.034 |
| Day-time SBP (mm Hg) | 0.05 (-0.01; 0.11) | 0.118 |
| Day-time DBP (mm Hg) | 0.02 (-0.08; 0.12) | 0.691 |
| Night-time SBP (mm Hg) | 0.02 (-0.04; 0.08) | 0.514 |
| Night-time DBP (mm Hg) | -0.02 (-0.10; 0.07) | 0.671 |
|  |  |  |
| Hypertension | -0.41 (-2.08; 1.27) | 0.619 |
| Antihypertensive drugs | 0.32 (-1.16; 1.79) | 0.657 |
| Dyslipidaemia | 0.74 (-1.06; 2.53) | 0.402 |
| Lipid lowering drugs | 1.23 (-0.09; 2.56) | 0.066 |
| MetS | 0.04 (-1.48; 1.57) | 0.954 |
| ESS-Epworth Sleepiness Scale; AHI-apnea/hypopnea index; ODI-oxygen desaturation index; SaO_2_-arterial oxygen saturation; SaO_2_<90%-time under 90%; HDL-high density lipoprotein; LDL-low density lipoprotein; SBP-systolic blood pressure; DBP-diastolic blood pressure; Dt-day-time; Nt-night-time; MetS-metabolic syndrome; cf-PWV-carotid-femoral pulse wave velocity. ∆cf-PWV = cf-PWV before CPAP- cf-PWV after CPAP. | | |
